# Effectiveness of a cognitive behavioural therapy (CBT)-based intervention for reducing anxiety among adolescents in the Colombo District, Sri Lanka: cluster randomized controlled trial

**DOI:** 10.1186/s13034-024-00799-9

**Published:** 2024-08-31

**Authors:** Sinha De Silva, Renuka Peris, Sudharshi Senaviratne, Dulani Samaranayake

**Affiliations:** 1 PGIM, University of Colombo, Colombo, Sri Lanka; 2 Ministry of Education (Retired), Colombo, Sri Lanka; 3 The Central Queensland Hospital and Health Service (CQHHS), Queensland, Australia; 4 Faculty of Medicine, University of Colombo, Colombo, Sri Lanka

## Abstract

Anxiety disorders are found to be the most prevalent psychological problems among children and adolescents. Cognitive behaviour therapy (CBT) was found to be effective at reducing anxiety. The purpose of this study was to assess the effectiveness of a universal school-based intervention for reducing anxiety among Grade 9 schoolchildren. A randomized controlled cluster trial was conducted by randomly assigning 36 schools in the Colombo district in Sri Lanka into study and control arms, each comprising 18 schools with 360 students. The levels of anxiety, self-esteem and depression status were assessed using the validated Screen for Child Anxiety Related Disorders (SCARED) tool and the Rosenberg self-esteem scale, respectively, and the Depression, Anxiety and Stress Scale 21 (DASS-21) at baseline, after intervention, and after 3 months. A CBT-based universal intervention package was administered weekly by a trained teacher for eight weeks with a one-month self-practice period to a randomly selected Grade 9 class in each school in the study arm. The control arm received routine care. Anxiety and self-esteem scores and depression status were compared between the two arms after the intervention and at 3 months of follow-up using the generalized estimation equation (GEE), controlling for confounding and clustering. The nonresponse and loss to follow-up rates were < 1%. When comparing the study arm with the control arm using GEE, anxiety levels were significantly lower [β = (-0.096), 95% CI = (-0.005) − (-0.186), *p* = 0.038] at follow-up but not postintervention [β = (-0.024), 95% CI = 0.006 − (-0.055), *p* = 0.115]. There were no significant differences in depression status after intervention (OR = 0.257, 95% CI =0.052–1.286; *p* = 0.098) or follow-up (OR = 0.422, 95% CI 0.177–1.008; *p* = 0.052), and self-esteem significantly increased after intervention (β = 0.811, 95% CI = 0.314–1.309; *p* = 0.001) but not at follow-up [β=0.435, 95% CI=(-0.276)-1.145, p=0.231]. This study revealed that the universal package based on CBT is effective at reducing anxiety and improving self-esteem among adolescents. The trial registration number and date were SLCTR/2018/018 and 19th of June 2018 respectively.

**Supplementary Information:**

The online version contains supplementary material available at 10.1186/s13034-024-00799-9.

## Introduction

Anxiety is defined as the anticipation of upcoming threats and is associated with muscle tension and vigilance in preparation for future danger and continuous or avoidant behavior [1]. It is recognized as a normal adaptive response to a stimulus that one will try to avoid. It is a brain response to something that is happening or might happen in the future. Anxiety becomes pathological, regardless of the individual’s age, when it is persistent or excessive and associated with subjective distress or impairment [2]. Individuals with any anxiety disorder (AD) may exhibit similar psychological, physical, and behavioral characteristics. These disorders are categorized based on the object or situation that induces fear, anxiety, and/or distress and avoidance behavior [2].

Anxiety disorders are the most prevalent form of psychopathology among adolescents [3–5]. Despite notable variations in prevalence estimates, likely due to methodological differences, the lifetime prevalence of ‘any anxiety disorder’ in studies with children or adolescents is approximately 15–20%. Period prevalence estimates, such as 1-year or 6-month rates, are not considerably lower than lifetime estimates [2]. A longitudinal study revealed significant linear associations between having anxiety disorders in adolescence and developing a range of adverse outcomes in early adulthood, including mental health problems like major depression, nicotine dependence, alcohol dependence, illicit drug dependence, and suicidal behavior [6].

Behavioral therapy plays a pivotal role in preventing and managing anxiety disorders. A review by Compton et al. (2004) of 21 randomized controlled trials concluded that cognitive behavioral therapy (CBT) is the treatment of choice for anxiety in children and adolescents, showing medium to large effect sizes for symptom reduction. CBT is favored due to its lack of adverse side effects, withdrawal problems, and association with a lower rate of subsequent relapse. Additionally, CBT can enhance self-esteem and foster an increased sense of agency [7, 8].

Anxiety prevention measures can be directed at individuals or groups. Evidence suggests that group-targeted preventive measures are more effective than those targeting individuals. Prevention programs targeting a group may be universal, selective, or indicated. Universal interventions are directed at the entire population regardless of risk status. Selective interventions involve people identified as at risk for psychological problems, while indicated interventions target those identified as having mild to moderate symptoms. Universal prevention strategies include elements of primordial, primary, secondary, and tertiary prevention, whereas selective programs focus on primary prevention, and indicated programs concentrate on secondary prevention [9].

There are several benefits associated with universal prevention programs compared to selective and targeted programs. Universal interventions can address people with limited access to treatment, ensuring no one is omitted. People with limited access to treatment show low dropout rates, which can help avoid the stigma associated with participating in selective or targeted interventions [9]. A systematic review of universal interventions for reducing anxiety and depressive symptoms in school-aged children revealed small but significant effects on reducing these symptoms. The goal of prevention is to reduce the likelihood of future negative outcomes by reducing relevant risk factors and strengthening protective factors [10]. When developed as prevention programs, these programs are designed to build skills rather than provide therapy, meaning strategies are learned for common situations rather than specific individual difficulties [11].

Despite the established effectiveness of school-based interventions for reducing anxiety, there is a scarcity of evidence, particularly in Southeast Asia. The importance of this study becomes increasingly evident when considering the unique interaction between interventions designed to reduce anxiety and contextual factors influencing their efficacy. These factors play a central role, particularly in educational settings characterized by distinct regional attributes. The educational environments of our region have unique characteristics that can significantly impact the effectiveness of these interventions. Therefore, it is crucial to conduct this intervention study tailored to our specific context. By doing so, we can gain a deeper understanding of how these unique environmental characteristics influence anxiety reduction strategies, allowing us to fine-tune the intervention in a contextually relevant manner.

This study was conducted to assess the effectiveness of a universal CBT-based intervention for reducing anxiety among adolescents in Sri Lanka. The study was carried out in the Sri Jayewardenepura Education Zone in the Colombo district, targeting Grade 9 schoolchildren. We employed a randomized controlled cluster trial, randomly assigning 36 schools into intervention and control arms. The intervention, delivered by trained teachers, consisted of eight weekly CBT sessions followed by a one-month practice period. Outcomes were assessed using validated tools at baseline, post-intervention, and three months follow-up.

By focusing on the effectiveness of a universal CBT-based intervention within the unique cultural and educational context of Sri Lanka, this study aims to provide insights into how contextual factors influence anxiety reduction strategies, thereby enhancing the relevance and applicability of the intervention in similar settings.

## Materials and methods

### Study design and participants

We conducted a randomized controlled multicenter trial in the Sri Jayewardenepura Education Zone in the Colombo district, Sri Lanka. The target population was children in grade nine (aged 13–15 years). Each school was considered a cluster, and a randomly selected class of nine grades was selected from each school for the study. The average number of students per group was 20. The sample size and number of groups for each arm were decided using a formula proposed by Hayes Moulton (2017) for an individually randomized trial with inflation for the design effect for a cluster randomized trial [12]. The values for the true mean and standard deviation of the outcome variable in the presence (µ_1_ = 7.35, σ_1_ = 6.93) and absence (µ_0_ = 9.58, σ_0_ = 6.44) of intervention were informed by Barrett and Turner (2001) in their study on reducing anxiety using a CBT-based package. For alpha (Z_α_) and beta (Z_β_) errors, the values used were Z_α_ = 1.96 for α = 0.05 and Z_β_ = 0.84 for β = 0.80 [13]. According to Shackleton et al. (2016), the ICC for psychological outcomes ranges from 0.01 to 0.07; we used the higher end of this range, setting ICC (ρ) at 0.07 to maximize sample size [14]. All Sinhala medium schools with year nine classes were invited to participate in the study; 37 schools agreed upon the study, and one school withdrew prior to randomization. A total of 720 students from 36 schools and their parents provided assent and informed written consent, respectively.

The trial was registered in the Sri Lanka Clinical Trial Register (SLCTR/2018/018) and was approved by the Ethics Review Committee of the Faculty of Medicine, University of Kelaniya (P/19/01/2018). Administrative approval was obtained from the Ministry of Education, Sri Lanka.

### Randomization and masking

**Fig. 1:**
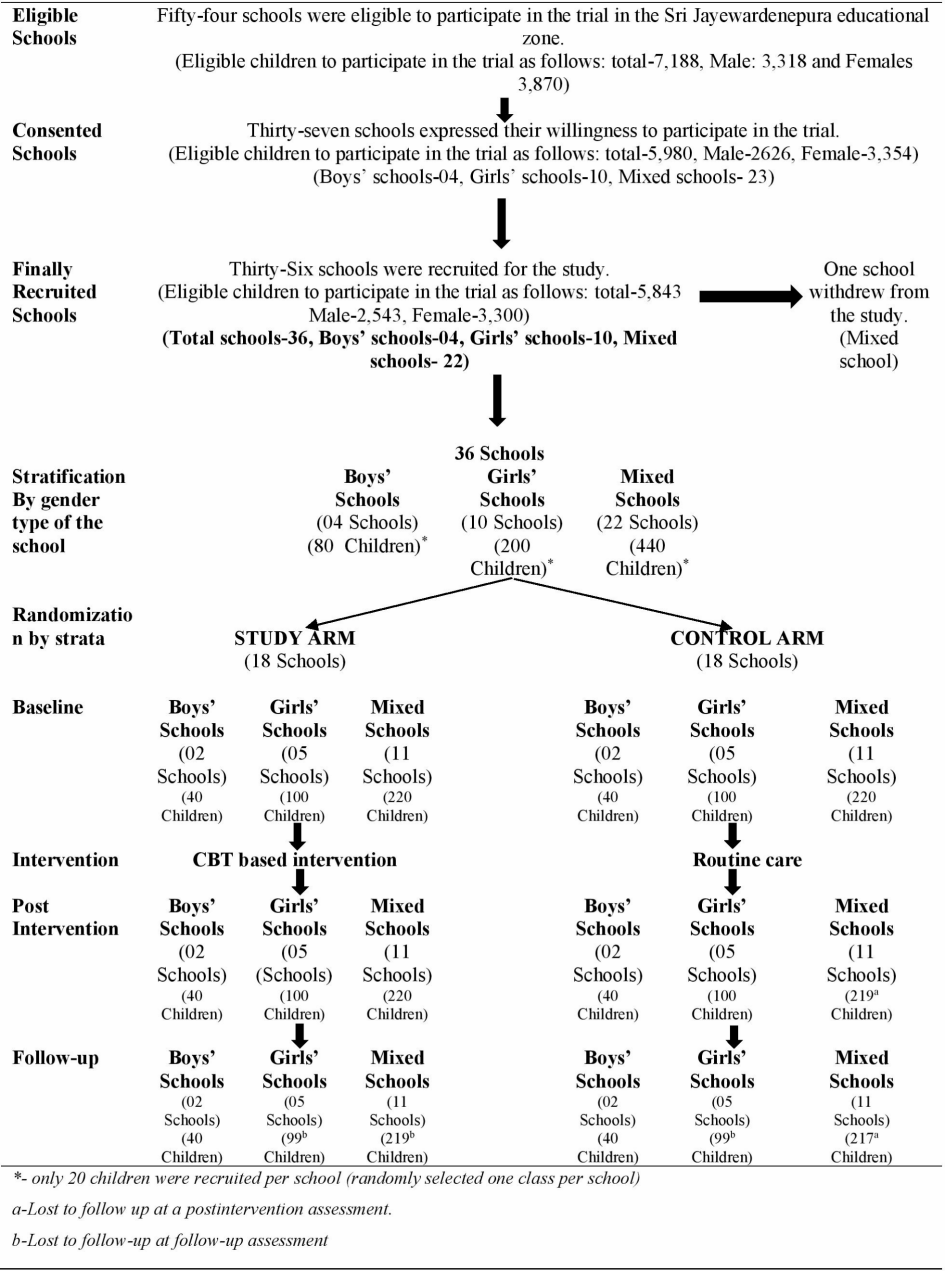
Student progress through the trial– CONSORT flow chart

The consented schools were stratified by school gender type, i.e., male, female, or mixed. Within each stratum, schools were randomized to the universal intervention group based on CBT or the control group using the block randomization method to ensure allocation of schools in a 1:1 ratio to two groups (Fig. 1). Schools, students, and the research team were masked in the allocation.

### Procedure

#### Intervention arm

Schools that were randomized into the intervention arm received a universal CBT-based intervention package that was developed based on the UK Medical Research Council guidance by De Silva et al. [15]. The intervention was delivered by a teacher who underwent a comprehensive training program covering CBT principles, specific techniques, and strategies for engaging students. To ensure consistency, the training included role-playing exercises, peer reviews, and feedback sessions. Teachers were provided with a detailed handbook outlining session plans and activities. The delivery of the intervention was monitored using a fidelity monitoring framework involving regular supervision by experienced mental health professionals. Teachers submitted weekly reports, and random observations were conducted to assess adherence to the intervention protocol. Any deviations were addressed through additional support and training, aiming to minimize variations and maintain the integrity of the intervention across different schools. Each teacher delivered the intervention package to randomly selected classes in the intervention arm in a 40-minute session per week for eight consecutive weeks and for a subsequent one-month duration to practice what they learned. An additional session was arranged for students who missed a session prior to the next session. During these eight sessions, the children were trained in CBT-based skills that were aimed at reducing anxiety and relaxing. The outline of the intervention package is shown in Table 1, and its details are published elsewhere by De Silva et al. (2023) [15]. They were instructed to practice these skills under the close and distant supervision of the teacher.

A 40-minute session was held for parents by the same teachers, overlapping with the routine parent meeting. Parents were educated about anxiety, anxiety disorders, how to identify them, and how to adapt to reduce anxiety. This session aimed to support parents in practicing the anxiety reduction skills learned by their children at home. Materials included the Teachers’ Handbook, the Workbook for Children, and a monitoring tool for students’ practice, along with a leaflet for parents. The intervention was monitored using the process evaluation framework (PEF) at the school level [15]. The PEF of the intervention group is provided in Additional File 1 Table S1.

**Table 1:** Outline of the content of the intervention delivered by the teacher

Session	Content
Within two weeks prior to commencement of intervention	Baseline assessment
Session 01 (Week 1)	-Psychoeducation on -Recognition and understanding emotions and feelings -What is anxiety?
Session 02 (Week 2)	-Training to avoid negative thoughts by understanding thoughts and feelings -Introduction and practising of relaxation exercises
Session 03 (Week 3)	-Training to avoid negative thoughts by understanding thoughts and feelings with scenario-based exercises. -practising of relaxation exercises
Session 04 (Week 4)	-Introduction of problem-solving skills -practising of relaxation exercises
Session 05 (Week 5)	-Problem solving skills with practical exercises -practising of relaxation exercises
Session 06 (Week 6)	- Improve skills on facing challenges (Graded exposure) -practising of relaxation exercises
Session 07 (Week 7)	-Practising of improve skills on facing challenges (Graded exposure) -practising of relaxation exercises
Session 08 (Week 8)	-Development of self-confidence -Revising of learned skills -practising of relaxation exercises
Session for parents (Within first eight weeks)	Psychoeducation on What is anxiety? Its harmful effects Steps to be followed to reduce anxiety
Week 9 and 12	Self-practice sessions
Postintervention assessment	Within two weeks from end of three months of the intervention
Follow up assessment	Within two weeks of completion of three months since postintervention assessment

We acknowledge the potential for performance bias due to the lack of blinding among intervention deliverers and participants. While blinding presents challenges in school-based interventions, several measures were taken to minimize bias and ensure the study’s internal validity. The rigorous training, monitoring procedures, and use of validated assessment tools, such as the SCARED tool for anxiety and the Rosenberg self-esteem scale, helped reduce subjective bias. Despite the difficulties in implementing blinding, these steps significantly contributed to the robustness of our findings. Future studies should explore feasible blinding strategies to further strengthen internal validity.

#### Control arm

Routine schoolwork was carried out in randomly selected classes from the control arm. The participants were assessed at baseline, after the intervention, and at follow-up.

### Outcomes and measures

Both groups were assessed at baseline using a self-administered questionnaire consisting of sociodemographic questions, a tool to measure the level of anxiety, the state of depression, and the level of self-esteem. The participants’ level of anxiety, depression status and self-esteem were assessed postintervention, as well as at three months of follow-up, using the same tools as follows.

#### Level of anxiety

The child version of the Screen for Child Anxiety Related Disorders (SCARED) is a 41-item instrument rated on a 3-point Likert scale. This instrument has been validated for assessing children aged 8–18 years [16, 17]. The translated and validated questionnaire, which has high reliability according to Cronbach’s alpha (0.87) and test-retest correlation coefficient (0.74), was used to assess the level of anxiety [18]. A higher score indicates a greater level of anxiety. 

#### Status of depression

Depression scales of the validated DAS-21 questionnaire were used in the local setting among adolescents by Weerasinghe in 2012. This approach has been validated for use in Sinhalese adolescents, as it has good psychometric properties. All the subscales had good reliability (Cronbach’s alpha > 0.7). Its cut-off value for the depression subscale was 19, with a sensitivity = 80% and specificity = 83%. [19]. The state of depression was determined using this tool.

#### Self-esteem

The Rosenburg self-esteem instrument was validated for use among adolescents internationally. The NRS-2002 is a 10-item scale that measures global self-worth by measuring both positive and negative feelings about the self. The scale is believed to be unidimensional. All the items are answered using a four-point Likert scale ranging from strongly agreeing to strongly disagreeing. The scores were on a continuous scale ranging from 10 to 40. A higher score indicates greater self-esteem [20, 21].

### Statistical analysis

All the analyses were based on intention-to-treat principles. All baseline categorical variables are presented as numbers and percentages, and all numerical variables are presented as the means and standard deviations (Table 2). As the first step of the pre specified statistical analysis plan (SAP), all the outcome variables were compared between two groups using conventional statistics with their effect sizes. Those results are presented in the (Additional File 1 Table S2 to Table S4).

The outcome variables were analysed to determine the effectiveness of the intervention using marginal linear regression with coefficients estimated by the generalized estimating equation (GEE). This approach allowed us to control for the effect of clustering and adjust for imbalanced covariates and probable confounders identified at the beginning of the study. Specifically, we considered several covariates and confounding variables, including sex, age, ethnicity, religion, permanent and current residence, school functional type, attendance at tuition classes, engagement in extracurricular activities, having siblings, anxiety sensitivity, behavioral inhibition, mother’s level of education, mother’s and father’s occupation, and perceived parenting style of the mother. By adjusting for these covariates, we aimed to isolate the effect of the intervention from other factors that might affect anxiety, depression, and self-esteem levels among the participants, thereby minimizing bias and improving the internal validity of our findings. The marginal models allow the effect of the explanatory variables on the outcome, and the correlation between observations is modelled separately. Each outcome variable was analysed after the intervention and at the follow-up; in other words, separate marginal models were used for each outcome variable after the intervention and at the follow-up. Only the relevant anxiety levels from the outcome tables are shown here (Tables 3 and 4), and the anxiety levels for depression status and self-esteem are shown in the supplementary material. In each marginal model, the scores of the outcome variable were used as the dependent variable, the arm of the study (intervention or control), the baseline value of the outcome variable and the baseline covariates that needed to be adjusted were used as predictors. Clusters were used as subjects to control for the nested/cluster effect. SPSS 22 was used for the data analysis.

## Results

Thirty-six clusters were recruited, and all the clusters remained throughout the study. Clusters were randomized into intervention and control arms as shown in Fig. 1. At the initial assessment before the beginning of the study, there were 720 students in both arms. At the postintervention assessment, only one student in the control arm was lost to follow-up, which was a 99.86% (*n* = 719) response rate. At the end of the three-month follow-up, only two students were lost to follow-up in the intervention arm, and four were lost to follow-up in the control arm (99.16%; *n* = 714). At both points of measurement, less than 0.9% of the patients were lost to follow-up. A comparison of the baseline sociodemographic and other selected characteristics between the study arm and the control arm was performed as follows (Table 2):

**Table 2:** Comparison of selected characteristics of the individuals in the study arm and control arm

Characteristic	Intervention arm	Control arm
**Sex**, n(%)		
Male	159 (44.2)	138 (38.3)
Female	201 (55.8)	222 (61.7)
**Age**, n(%)		
13 years	155 (43.1)	166 (46.1)
14 years	205 (56.9)	194 (53.9)
**Ethnicity**, n(%)		
Sinhala	351 (97.5%)	346 (96.1)
Tamil	02 (0.6)	0 (0.0)
Muslim	05 (1.3)	13 (3.6)
Burgher	02 (0.6)	01 (0.3)
**Religion**, n (%)		
Buddhism	347 (96.4)	342 (95.0)
Christianity	08 (2.2)	5 (1.4)
Islam	05 (1.4)	13 (3.6)
**Status of permanent residence**, n (%)		
Within Colombo district	354 (98.3)	346 (96.1)
Out of Colombo district	06 (1.7)	14 (3.9)
**Status of current residence**, n (%)		
Staying away from the family	16 (4.4)	09 (2.5)
**Staying with the family**	344 (95.6)	351 (97.5)
Functional type of school attending		
Type 1AB	192 (53.3)	220 (61.1)
Type 1 C	75 (20.8)	80 (22.2)
Type 2	93 (25.8)	60 (16.7)
**Status of attending tuition classes**		
Yes	335 (93.1)	323 (89.7)
No	25 (6.9)	37 (10.3)
**Status of engaging in an extracurricular activity**, n (%)		
Yes	60 (16.7)	74 (20.6)
No	300 (83.3)	286 (79.4)
**Status of having siblings**, n (%)		
Yes	312 (86.7)	324 (90.0)
No	48 (13.3)	36 (10.0)
**Status of Anxiety Sensitivity**, n (%)		
High	90 (25.0)	71 (19.4)
Moderate to low	270 (75.0)	289 (80.6)
**Status of Behavioural inhibition**, n (%)		
High	29 (8.1)	43 (11.9)
Moderate to low	331 (91.9)	317 (88.1)
**Mother’s level of education**, n (%)		
Yes	32 (8.9)	13 (3.6)
No	325 (90.1)	343 (96.4)
**Whether the mother is engaged in a foreign employment**, n (%)		
Yes	24 (6.7)	15 (4.2)
No	333 (93.3)	341 (95.8)
**Perceived parenting style of the mother**, n (%).		
Rejection	10 (2.8)	20 (5.6)
Emotional warmth	130 (36.3)	111 (31.2)
Over protection	218 (60.9)	225 (63.2)
**Whether the father is engaged in a foreign employment**, n (%)		
Yes	01 (0.3)	11 (3.1)
No	345 (99.7)	343 (96.9)
**SCARED Child score total score**, mean (SD)	26.0 (12.5)	27.2 (11.4)
**DASS 21 Depression scale score**, mean (SD)	7.6 (8.6)	7.4 (7.9)
**Rosenberg Self-esteem scale score**, mean (SD)	30.0 (3.92)	29.7 (1.1)

### Level of anxiety

The standardized mean difference (Cohen’s d) between the levels of anxiety in the two groups at post-intervention was calculated to be 0.12. The estimated regression coefficients of a marginal model with robust standard error to show the effects of the intervention arm on the outcome, the SCARED Child Sinhala score postintervention, after adjustment for the baseline SCARED Child score and other selected covariates/factors while controlling for the clustering effect are shown in Table 3.

**Table 3:** Estimates of the marginal model for the effect of the intervention on the SCARED child Sinhala score postintervention after adjustment for confounders and the clustering effect

Parameter	Beta	Std. Error	95% Wald Confidence Interval		Hypothesis Test		
			Upper	Lower	Wald Chi-Square	Df	*p* value
(Intercept)	2.000	0.0792	1.845	2.155	637.291	1	0.001
**Arm: Study Arm**	**-0.024**	**0.0155**	**-0.055**	**0.006**	**2.488**	**1**	**0.115**
Sex: Female	0.03	0.0137	0.057	0.004	4.919	1	0.027
Ethnicity: Non Sinhala	0.079	0.1403	-0.196	0.354	0.317	1	0.574
Religion: Non Buddhist	-0.131	0.1306	-0.387	0.125	0.999	1	0.318
Permanent residence: Out of Colombo	0.016	0.0335	-0.049	0.082	0.236	1	0.627
Leadership: Never	0.039	0.0156	0.07	0.009	6.279	1	0.012
School functional type 2 compared to 1AB	-0.007	0.0211	-0.048	0.035	0.103	1	0.748
School functional type C compared to 1AB	-0.027	0.0156	-0.057	0.004	2.982	1	0.084
Attend tuition: No	0.041	0.019	0.004	0.079	4.752	1	0.029
Behavioral Inhibition: High	0.029	0.0237	-0.018	0.075	1.447	1	0.229
Anxiety Sensitivity: High	0.049	0.0146	0.021	0.078	11.392	1	0.001
Mother’s parenting style: overprotection compared to Rejection	0.046	0.0322	-0.017	0.109	2.03	1	0.154
Mother’s parenting style: Emotional warmth compared to Rejection	0.026	0.0386	-0.05	0.102	0.456	1	0.499
Father’s occupation: Not foreign employment	0.008	0.0282	-0.047	0.063	0.084	1	0.772
Mother’s occupation: Not foreign employment	0.051	0.028	-0.003	0.106	3.373	1	0.066
Mother’s level of education: More than primary education	-0.018	0.0313	-0.08	0.043	0.341	1	0.559
Baseline: SCARED Child score	0.041	0.0013	0.038	0.043	955.69	1	< 0.001

Bold values indicate the effect of the intervention compared to the control arm, adjusted for the variables listed in the table and accounting for clustering

Dependent variable: anxiety level: SCARED child score at postintervention

Model: Intercept, arm, sex, ethnicity, religion, permanent residence, leadership, school functional type, attended tuition, behavioural inhibition, anxiety sensitivity, mother’s parenting style, father’s occupation, mother’s occupation, mother’s level of education, baseline: SCARED Child score

The clustering effect is adjusted in GEE by considering clusters as the subject while analysing individual-level data

The standardized mean difference (Cohen’s d) between the levels of anxiety in the two groups at follow-up was calculated to be 0.32.The estimated regression coefficient of a marginal model with robust standard error was used to show the effects of the intervention arm on the outcome, the SCARED Child score at follow-up, after adjustment for the baseline SCARED Child score, and other selected covariates/factors controlling for the clustering effect (Table 4).

**Table 4:** Estimates of the marginal model for the effect of the intervention on the SCARED child Sinhala score at follow-up after adjustment for confounders and the clustering effect

Parameter	Beta	Std. Error	95% Wald Confidence Interval		Hypothesis Test		
			Upper	Lower	Wald Chi-Square	Df	*p* value
(Intercept)	2.01	0.1086	1.797	2.223	342.531	1	< 0.001
**Arm: Study Arm**	**-0.096**	**0.0461**	**-0.186**	**-0.005**	**4.311**	**1**	**0.038**
Sex: Female	0.054	0.0235	0.1	0.008	5.244	1	0.022
Ethnicity: Non-Sinhala	0.138	0.1493	-0.154	0.431	0.859	1	0.354
Religion: Non-Buddhist	-0.149	0.1245	-0.393	0.095	1.44	1	0.230
Permanent residence: Out of Colombo	0.041	0.0236	-0.005	0.087	3.024	1	0.082
Leadership: Never	0.055	0.0202	0.094	-0.015	7.326	1	0.007
School functional type 2 compared to 1AB	-0.001	0.0557	-0.11	0.108	0.001	1	0.98
School functional type C compared to 1AB	0.041	0.0611	-0.079	0.161	0.446	1	0.504
Attend tuition: No	0.041	0.019	0.004	0.078	4.613	1	0.032
Behavioral Inhibition: High	0.055	0.0205	0.015	0.095	7.199	1	0.007
Anxiety Sensitivity: High	0.055	0.0174	0.021	0.089	9.901	1	0.002
Mother’s parenting style: overprotection compared to Rejection	0.021	0.0336	-0.045	0.087	0.4	1	0.527
Mother’s parenting style: Emotional warmth compared to Rejection	-0.003	0.0317	-0.065	0.059	0.008	1	0.928
Father’s occupation: Not foreign employment	0.009	0.0467	-0.083	0.1	0.036	1	0.849
Mother’s occupation: Not foreign employment	0.097	0.0304	0.037	0.156	10.154	1	0.001
Mother’s level of education: More than primary education	0.008	0.0385	-0.067	0.083	0.042	1	0.837
Baseline: SCARED Child score	0.038	0.0014	0.035	0.041	743.667	1	< 0.001

Bold values indicate the effect of the intervention compared to the control arm, adjusted for the variables listed in the table and accounting for clustering

Dependent variable: Anxiety level: SCARED Child score at follow-up

Model: Intercept, arm, sex, ethnicity, religion, permanent residence, leadership, school functional type, attended tuition, behavioral inhibition, anxiety sensitivity, mother's parenting style, father’s occupation, mother’s occupation, mother’s level of education, baseline: SCARED child score

The clustering effect is adjusted in GEE by considering clusters as subjects while analysing individual-level data

### Status of depression

The standardized mean differences (Cohen’s d) in depression levels between the intervention and control groups were 0.04 at post-intervention and 0.06 at follow-up. The generalized estimating equation was used with clusters as the subject variables, a robust estimator as the covariance matrix, AR as the working correlation matrix structure, a binomial logit as the ordinal response under the type of model, and predictors that included the arm of the intervention, baseline DASS-21 Depression Scale score at baseline and some selected covariates. The status of depression, that is, having or not having depression at the postintervention time point and follow-up, was assessed as the dependent variable in two separate models (Additional File 1 Table S5 and Table S6). The results suggest that the study arm participants had lower odds of having depression after the intervention time point, with an OR of 0.257 (95% CI 0.05–1.27) and an OR of 0.422 (95% CI 0.177–1.008) at the follow-up time point than did the control arm participants; however, these differences were not statistically significant, with *p* = 0.098 and *p* = 0.052, respectively, when adjustments were made for imbalance correlates/confounders at baseline and for the effect of clustering in two separate models.

### Level of self-esteem

The standardized mean differences (Cohen’s d) in self-esteem levels between the intervention and control groups were 0.3 at post-intervention and 0.2 at follow-up. The regression coefficients were estimated from two different marginal models with robust standard errors to show the effects of the intervention on the Rosenberg self-esteem scale scores after the intervention and at follow-up after adjustment for the Rosenberg self-esteem scale scores at baseline and other selected covariates/factors while controlling for the cluster effect (Additional File 1 Table S7 and Table S8). The results suggest that the participants in the study arm had significantly greater Rosenberg self-esteem scale scores at the postintervention time point (i.e., high self-esteem) (0.811 points; 95% CI = 0.314 to 1.309, *p* = 0.001) than did the participants in the control arm; however, the participants in the study arm had greater Rosenberg self-esteem scale scores at the follow-up time point (i.e., high self-esteem) [0.435; 95% CI = (-0.276) to 1.145] than did the participants in the control arm did, but it was not statistically significant, as the p value was 0.231 when adjustments were made for imbalanced correlates at baseline and for the effect of clustering.

## Discussion

We examined the effectiveness of teacher-provided universal CBT-based interventions for reducing anxiety among adolescents in a school setting. The loss to follow-up was minimal, and good compliance with the intervention was reported. The baseline variables were comparable between the two arms.

There was a reduction in the level of anxiety (SCARED Child score) after the intervention and at the follow-up, but it was statistically significant only at the follow-up in the intervention arm compared to the control arm. Depression status was also reduced in the intervention arm than in the control arm at both postintervention and follow-up. The level of self-esteem (the Rosenberg self-esteem tool score) was greater in the intervention arm than in the control arm at both postintervention and follow-up but was statistically significant only at postintervention assessment.

A systematic review by Corrieri et al. 2014 of 28 RCTs conducted after the year 2000 in which outcomes were measured as continuous variables with self-reported instruments, focusing on school-based prevention interventions for anxiety and depression with the aim of exploring their effectiveness, revealed that 15 studies [10] were reported to be effective (73%), and 16 (67%) out of 24 studies reported effective outcomes for anxiety and depression [22]. Furthermore, Werner-Seidler et al. (2017) revealed that psychological program-based interventions are effective at reducing anxiety and depression after intervention and at follow-up in a systematic review that included 81 studies with 31,794 participants [23]. Similar findings to those of the present study were explored in a cluster randomized controlled trial with a universal school-based intervention based on CBT administered in 10 weekly sessions among 638 children aged 9–12 years in 14 schools in Germany, where anxiety level was assessed using a self-reported instrument (SCAS). A statistically significant reduction in anxiety was observed at follow-up (*p* < 0.05) but not postintervention [24].

Despite the fact that CBT-based interventions were effective at significantly reducing the level of depression, as shown by systematic reviews, the present study did not show a significant effect on reducing depression. Furthermore, a reduction in the level of depression was shown in the intervention arm, and a follow-up arm comparison became nonsignificant at *p* = 0.52. These differences may be due to an inadequate sample size for detecting changes with respect to the level of depression.

Like in the current study, a CBT-based RCT conducted by Amin et al. (2020) among 76 adolescents aged 13–16 years in Pakistan with a study arm and a control arm, with the objective of evaluating its effectiveness in increasing self-esteem using the Rosenberg self-esteem scale for outcome evaluation, revealed a statistically significant increase in self-esteem levels in the study arm compared to the control arm (*p* < 0.001) [25].

Therefore, it could be concluded that the findings of the present intervention study align with the global literature. Furthermore, from a practical point of view, the importance of this study is manifold. First and foremost, it was carried out by the teachers themselves, seamlessly integrating into the existing educational framework. This approach not only emphasizes the feasibility of the study but also highlights its minimal intrusion into the regular schedule, preserving the quality of academic hours. In addition, the enthusiastic participation of students serves as a testament to the study’s engagement and relevance to their needs. The fact that the tool was well received by the student body not only adds to its credibility but also demonstrates its practical value in real-world educational settings. In essence, this study provides an example of how educational research can be conducted effectively and efficiently, with direct benefits for both educators and students. Therefore, universal CBT-based interventions are effective at reducing anxiety and increasing self-esteem among schoolchildren in Sri Lanka.

In order to address the delay in achieving statistical significance, it is important to consider multiple contributing factors. While the time taken by participants to practice learned skills is a key element, other potential influences must be acknowledged. Individual differences in response to the intervention, such as varying levels of baseline anxiety and differing capacities for skill acquisition and application, likely played a role. Contextual factors, including the school environment and external stressors, may have impacted the effectiveness of the intervention. Additionally, methodological limitations, such as potential biases in self-report measures and the challenges of maintaining consistency across different school settings, could have influenced the outcomes. Recognizing these factors provides a more comprehensive understanding of the intervention’s effectiveness over time and highlights areas for future research to further optimize CBT-based interventions in similar contexts. Also, in view of addressing the discrepancy in detecting changes in depression levels, several factors need to be considered. The intensity and duration of the intervention might not have been sufficient to produce a measurable effect on depression within the study period. Future research should explore longer intervention durations, increased intensity, and tailored approaches to address depression more effectively in similar populations.

The observed improvement in self-esteem among participants in the intervention arm can be attributed to several factors inherent in CBT techniques, which are particularly relevant within the cultural and educational context of Sri Lanka. CBT emphasizes the identification and restructuring of negative thought patterns, leading to a more positive self-view and enhanced self-esteem. In the Sri Lankan context, where students often face high academic pressures and societal expectations, learning to manage stress and develop a healthier cognitive outlook can significantly impact their self-esteem. Additionally, the group-based nature of the intervention facilitated peer support and interaction, providing a sense of community and shared experience among students. This is particularly important in the collectivist culture of Sri Lanka, where community and social relationships play a crucial role in individual well-being. The involvement of teachers, who are respected authority figures, in delivering the intervention likely contributed to its effectiveness by reinforcing the importance and credibility of the skills being taught. Future research should continue to explore these dimensions to further optimize the effectiveness of CBT-based interventions in diverse settings.

## Conclusions

Universal CBT-based interventions delivered by schoolteachers are effective at reducing anxiety and increasing self-esteem among schoolchildren. The observed improvements, although statistically significant, reflect a modest effect size, indicating that while the intervention is beneficial, its impact may vary among individuals. The sustainability of these effects over the long term remains a crucial area for further research, as continued practice and reinforcement of CBT skills are likely necessary to maintain these benefits.

From a practical standpoint, this approach can be easily incorporated into existing educational systems with minimal external resources, making it a feasible and scalable option for improving mental health at the school level. However, implementing such interventions in real-world settings presents challenges, including ensuring fidelity to the intervention protocol, providing adequate training and support for teachers, and addressing varying levels of resource availability across different schools. Future studies should focus on these aspects to enhance the practicality and effectiveness of CBT-based interventions in diverse educational contexts.

## Limitations

The study acknowledged several limitations. Performance bias due to the lack of blinding among intervention deliverers and participants was a potential issue, despite measures taken to minimize it. The sample size may have been inadequate to detect significant changes in depression levels. The delay in achieving statistical significance could be attributed to multiple factors, including the time taken by participants to practice learned skills, individual differences in response to the intervention, contextual factors such as the school environment and external stressors, and methodological limitations like potential biases in self-report measures and consistency challenges across different school settings. Additionally, the intensity and duration of the intervention might not have been sufficient to produce measurable effects on depression within the study period. The sustainability of the intervention’s effects over the long term remains uncertain, requiring continued practice and reinforcement of CBT skills. Finally, practical challenges in implementing such interventions in real-world settings include ensuring fidelity to the intervention protocol, providing adequate training and support for teachers, and addressing varying levels of resource availability across different schools.

### Electronic supplementary material

Supplementary Material 1.

## Data Availability

The data that support the findings of this study is available from the authors upon reasonable request.
