# Supplementary material for: Effectiveness of a cognitive behavioural therapy (CBT)-based intervention for reducing anxiety among adolescents in the Colombo District, Sri Lanka: cluster randomized controlled trial
Source: Child Adolesc Psychiatry Ment Health. 2024 Aug 31;18:108. doi: 10.1186/s13034-024-00799-9 (PMC11366126; doi:10.1186/s13034-024-00799-9)
Supplement: Supplementary file 1 — Supplementary Material 1. [file 13034_2024_799_MOESM1_ESM.docx]

Additional File

Table S1: The Process Evaluation Framework on Reach, Dose Delivered, Dose Received, Fidelity, Context and Participants Attitudes

|  | Indicator | | | N | | % | |
| --- | --- | --- | --- | --- | --- | --- | --- |
| Reach ^a^ | The number and the percentage of schools participated in the intervention out of randomized schools into the study arm | | | 18 | | 100.0 | |
|  | The number and the percentage of children recruited to the study out of the planned number of students to be recruited | | | 360 | | 100.0 | |
| Dose delivered ^b^ | Number and percentage of schools delivered all the planned sessions for children i.e.8 out of recruited schools for the intervention | | | 18 | | 100.0 | |
|  | Number and percentage of schools delivered the session for parents out of recruited schools for the intervention | | | 18 | | 100.0 | |
| Dose received ^b^ | Number and percentage of school children attended all eight sessions out of the recruited students | | | 299 | | 93.4 | |
|  | Number and percentage of school children attended at least seven sessions out of the recruited students | | | 316 | | 98.7 | |
|  | Number and percentage of school children attended at least six sessions out of the recruited students | | | 320 | | 100.0 | |
|  | Number and percentage of parents attended the sessions for parents (Total number of children recruited was taken as the denominator) | | | 229 | | 71.6% | |
|  | Number and percentage of teachers received teachers’ handbook | | | 320 | | 100.0 | |
|  | Number and percentage of children received the workbook | | | 320 | | 100.0 | |
|  | Number and percentage of leaflets sent to the parents | | | 320 | | 100.0 | |
| Fidelity ^b^ | % of schools completed the eight sessions for children in the stipulated period | | | 18 | | 100.0 | |
|  | % of schools completed the session for the parents in the stipulated period | | | 16 | | 88.9 | |
| Context ^c^ | | Support by the school management | Satisfied | | 18 | | 100.0 |
|  |  |  | Not Satisfied | | 0 | | 0.0 |
|  |  | Support by the immediate supervisors | Satisfied | | 18 | | 100.0 |
|  |  |  | Not Satisfied | | 0 | | 0.0 |
|  |  | Availability of infrastructure to facilitate the intervention | Satisfied | | 18 | | 100.0 |
|  |  |  | Not Satisfied | | 0 | | 0.0 |
|  | |  |  | |  | |  |
| Participants’ attitudes ^d^ | | Clarity of the study materials provided at the program | Satisfied | | 48 | | 96.0 |
|  | |  | Not Satisfied | | 02 | | 4.0 |
|  | | Understandability of the content of technical inputs given at the lectures | Satisfied | | 42 | | 84.0 |
|  | |  | Not Satisfied | | 08 | | 16.0 |
|  | | Overall time management at the program | Satisfied | | 39 | | 78.0 |
|  | |  | Not Satisfied | | 11 | | 22.0 |
|  | | The usefulness of the program | Satisfied | | 50 | | 100.0 |
|  | |  | Not Satisfied | | 0 | | 0.0 |

*a-This data was collected by PI at ToT workshop for teachers and at the commencement of the study.*

*b-These indicators were monitored at individual school level. Then those findings were summated and presented in this table.*

c- *This data was collected after completion of the intervention from the 18 teachers who delivered the intervention.* *Each stem of the questionnaire was given four-point Likert scale*

*(1-Strongly not satisfied, 2-Not satisfied, 3-Satisfied and 4-Strongly Satisfied). The responses marked as 1 and 2 were grouped as ‘not satisfied’ and 3 and 4 were grouped as ‘Satisfied’.*

*d- participants’ attitudes were assessed in a random sample of 50 students who were in the intervention arm. This data was collected following the completion of the intervention. Each stem of the questionnaire was given four-point Likert scale (1-Strongly not satisfied, 2-Not satisfied, 3-Satisfied and 4-Strongly Satisfied). The responses marked as 1 and 2 were grouped as ‘not satisfied’ and 3 and 4 were grouped as ‘Satisfied’.*

**Pre-specified statistical analysis plan (SAP) and results of the comparison of three main outcome variables between intervention and control group**

The pre specified statistical analysis plan (SAP) includes

Step 1

Each outcome measure was compared between intervention and control arm using conventional statistics. As the variables under study were skewed, the Mann-Whitney U test was used to do the comparison.

Furthermore, the effect size of the difference was assessed using ‘r’

The result of the conventional analysis is presented below all three main outcomes.

Step 2

Since we have used a cluster randomized controlled study , the results would be influenced by the clustering effect. Furthermore, confounding variables could distort the final result. In view of controlling for clustering effect and influence of the confounding variables, generalized estimation equation technique is used and presented in the manuscript.

Effectiveness of the intervention using conventional statistics.

Comparison of anxiety levels (SCARED Child scores) between study and control arms at post-intervention and follow up time points as follows (Table S2).

Table S2: Comparison of Anxiety Levels (SCARED Child Sinhala Scores) Between the Study Arm and the Control Arm at Post-Intervention and at Follow Up Time Points

| Time | Study Arm | | Control Arm | | Statistics^*^ | Effect size  r (z/√N) |
| --- | --- | --- | --- | --- | --- | --- |
|  | Median | IQR | Median | IQR | Z  p |  |
| Post-intervention | 25.0 | 13.0 | 26.0 | 15.0 | z=-1.749  p=0.08 | r=0.07 |
|  |  |  |  |  |  |  |
| Follow up | 23.0 | 14.0 | 26.0 | 17.0 | z=-4.148  p<0.001 | r=0.15 |

**-Mann-Whitney U Test was applied*

Comparison of depression levels (DASS-21 Depression scale scores) between study arm and control arm at post-intervention and follow up time points as follows (Table S3).

Table S3: Comparison of Depression Levels (DASS-21 Depression Scale Scores) Between the Study Arm and the Control Arm at Post-Intervention and at Follow-up Time Points

| Time | Study Arm | | Control Arm | | Statistics^*^ | Effect size  r (z/√N) |
| --- | --- | --- | --- | --- | --- | --- |
|  | Median | IQR | Median | IQR | Z  p |  |
| Post-intervention | 4.0 | 10.0 | 4.0 | 12.0 | z=-1.072  p=0.284 | r=0.04 |
|  |  |  |  |  |  |  |
| Follow up | 4.0 | 10.0 | 6.0 | 12.0 | z=-1.432  p=0.152 | r=0.05 |

^* Mann Whitney U test was applied^

Comparison of self-esteem levels (Rosenberg’s Self-esteem scale scores) between study arm and control arm at post-intervention and follow up time points as follows (Table S4).

Table S4: Comparison of Self-Esteem Scores (Rosenberg self-esteem scores) Between the Study Arm and the Control Arm at Post-Intervention and at Follow up Time Points (N=720)

| Time | Study Arm | | Control Arm | | Statistics^*^ | Effect size  r (z/√N) |
| --- | --- | --- | --- | --- | --- | --- |
|  | Median | IQR | Median | IQR | Z  P |  |
| Post-intervention | 30.0 | 6.0 | 29.0 | 6.0 | z=-2.397  p=0.017 | r=0.09 |
|  |  |  |  |  |  |  |
| Follow up | 31.0 | 8.0 | 30.0 | 5.0 | z=-2.030  p=0.042 | r=0.08 |

**Mann Whitney U test was applied*

Table S5: Estimates of the Marginal Model to Determine the Effects of the Intervention on the Status of Depression at Post-Intervention, After Adjustment for Confounding Factors and or Clustering Effect.

| Parameter | Exponential  (Beta) | 95% Wald Confidence Interval | | Hypothesis Test | | |
| --- | --- | --- | --- | --- | --- | --- |
|  |  | Upper | Lower | Wald  Chi-Square | df | p-value |
| **Arm: Study Arm** | **0.257** | **0.052** | **1.286** | **2.733** | **1** | **0.098** |
| Sex: Female | 0.421 | 0.099 | 1.788 | 1.373 | 1 | 0.241 |
| Ethnicity: Non- Sinhala | 1.418 | 0.3 | 6.713 | 0.194 | 1 | 0.66 |
| Permanent residence: Out of Colombo | 0.025 | -0.507 | 0.557 | 0.008 | 1 | 0.927 |
| Leadership: Never | 1.11 | 0.345 | 3.575 | 0.031 | 1 | 0.861 |
| School functional type 2 compared to 1AB | 1.418 | 0.3 | 6.713 | 0.194 | 1 | 0.66 |
| School functional type C compared to 1AB | 1.48 | 0.368 | 5.953 | 0.305 | 1 | 0.581 |
| Attend tuition: No | 0.328 | 0.054 | 1.98 | 1.478 | 1 | 0.224 |
| Behavioral Inhibition: High | 2.3 | 0.471 | 11.217 | 1.061 | 1 | 0.303 |
| Anxiety Sensitivity: High | 2.72 | 0.828 | 8.943 | 2.717 | 1 | 0.099 |
| Mother’s parenting style: overprotection compared to Rejection | 2.963 | 0.589 | 14.91 | 1.737 | 1 | 0.188 |
| Mother’s parenting style: Emotional warmth compared to Rejection | 1.972 | 0.262 | 14.85 | 0.435 | 1 | 0.51 |
| Father’s occupation: Not foreign employment | 1.313 | 0.479 | 3.598 | 0.28 | 1 | 0.597 |
| Mother’s occupation: Not foreign employment | 1.536 | 0.325 | 7.251 | 0.294 | 1 | 0.588 |
| Mother’s level of education: More than primary education | 0.485 | 0.035 | 6.768 | 0.29 | 1 | 0.59 |
| Baseline: DASS-21 Depression score | 1.612 | 1.437 | 1.81 | 65.976 | 1 | <0.001 |

*Dependent variable: Depression status at post-intervention*

*Model: Intercept, Arm, Sex, Ethnicity, Religion, Permanent residence, Leadership, School functional type, Attend tuition, Behavioral Inhibition, Anxiety Sensitivity, Mother’s parenting style, Father’s occupation, Mother’s occupation, Mother’s level of education, Baseline: DASS-21-Depression Child score*

*Clustering effect is adjusted in GEE by considering clusters as subject while analysis of individual-level data and the intercept was not included here*

Table S6: Estimates of the Marginal Model to Determine the Effects of the Intervention on the Status of Depression at Follow-up, After Adjustment for Confounding Factors and or Clustering Effect.

| Parameter | Exponential  Beta | 95% Wald Confidence Interval | | Hypothesis Test | | |
| --- | --- | --- | --- | --- | --- | --- |
|  |  | Upper | Lower | Wald  Chi-Square | Df | p-value |
| (Intercept) | 0.0001 | 0.00006 | 0.006 | 25.037 | 1 | <0.001 |
| **Arm: Study Arm** | **0.422** | **0.177** | **1.008** | **3.768** | **1** | **0.052** |
| Sex: Female | 1.292 | 0.352 | 4.745 | 0.149 | 1 | 0.700 |
| Ethnicity: Non- Sinhala | 1.948 | 0.22 | 17.229 | 0.36 | 1 | 0.549 |
| Religion: Non- Buddhist | -0.661 | -2.465 | 1.143 | 0.516 | 1 | 0.473 |
| Permanent residence: Out of Colombo | 0.054 | 0.003 | 0.929 | 4.043 | 1 | 0.044 |
| Leadership: Never | 1.069 | 0.361 | 3.167 | 0.014 | 1 | 0.905 |
| School functional type 2 compared to 1AB | 0.615 | 0.165 | 2.293 | 0.524 | 1 | 0.469 |
| School functional type C compared to 1AB | 0.84 | 0.191 | 3.692 | 0.053 | 1 | 0.818 |
| Attend tuition: No | 0.452 | 0.092 | 2.225 | 0.954 | 1 | 0.329 |
| Behavioral Inhibition: High | 0.754 | 0.169 | 3.357 | 0.137 | 1 | 0.711 |
| Anxiety Sensitivity: High | 3.399 | 0.869 | 13.294 | 3.091 | 1 | 0.079 |
| Mother’s parenting style: overprotection compared to Rejection | 2.57 | 0.735 | 8.98 | 2.186 | 1 | 0.139 |
| Mother’s parenting style: Emotional warmth compared to Rejection | 1.931 | 0.35 | 10.662 | 0.57 | 1 | 0.450 |
| Father’s occupation: Not foreign employment | -0.089 | -0.643 | 0.465 | 0.099 | 1 | 0.753 |
| Mother’s occupation: Not foreign employment | 1.358 | 0.31 | 5.958 | 0.165 | 1 | 0.685 |
| Mother’s level of education: More than primary education | 0.296 | 0.037 | 2.344 | 1.328 | 1 | 0.249 |
| Baseline: DASS-21 Depression | 1.503 | 1.385 | 1.63 | 96.344 | 1 | <0.001 |

*Dependent variable: depression status at follow up*

*Model: Intercept, Arm, Sex, Ethnicity, Religion, Permanent residence, Leadership, School functional type, Attend tuition, Behavioral Inhibition, Anxiety Sensitivity, Mother’s parenting style, Father’s occupation, Mother’s occupation, Mother’s level of education, Baseline: DASS-21-Depression scale score*

*Clustering effect is adjusted in GEE by considering clusters as subject while analysis of individual-level data*

Table S7: Estimates of the Marginal Model for the Effect of the Intervention on Rosenberg’s Self-Esteem Scale Score at the Post-Intervention, After Adjustment for Confounders and Clustering Effect.

| Parameter | Beta | Std. Error | 95% Wald Confidence Interval | | Hypothesis Test | | |
| --- | --- | --- | --- | --- | --- | --- | --- |
|  |  |  | Upper | Lower | Wald  Chi-Square | df | p-value |
| (Intercept) | 11.222 | 1.9897 | 7.323 | 15.122 | 31.813 | 1 | <0.001 |
| **Arm: Study Arm** | **0.811** | **0.2539** | **0.314** | **1.309** | **10.215** | **1** | **0.001** |
| Sex: Female | -0.072 | 0.2468 | -0.556 | 0.412 | 0.085 | 1 | 0.770 |
| Ethnicity: Non- Sinhala | -1.785 | 1.2312 | -4.198 | 0.629 | 2.101 | 1 | 0.147 |
| Religion: Non- Buddhist | 1.22 | 0.5811 | 0.081 | 2.359 | 4.407 | 1 | 0.036 |
| Permanent residence: Out of Colombo | -0.982 | 0.5199 | -2.001 | 0.037 | 3.564 | 1 | 0.059 |
| Leadership: Never | -0.119 | 0.2704 | -0.649 | 0.411 | 0.194 | 1 | 0.659 |
| School functional type 2 compared to 1AB | 0.004 | 0.0136 | -0.023 | 0.03 | 0.07 | 1 | 0.791 |
| School functional type C compared to 1AB | -0.002 | 0.0074 | -0.016 | 0.013 | 0.069 | 1 | 0.793 |
| Attend tuition: No | 0.759 | 0.5669 | -0.352 | 1.87 | 1.793 | 1 | 0.181 |
| Behavioral Inhibition: High | 0.147 | 0.3249 | -0.49 | 0.783 | 0.203 | 1 | 0.652 |
| Anxiety Sensitivity: High | -0.13 | 0.2699 | -0.659 | 0.399 | 0.232 | 1 | 0.630 |
| Mother’s parenting style: overprotection compared to Rejection | -0.659 | 0.5724 | -1.781 | 0.463 | 1.325 | 1 | 0.250 |
| Mother’s parenting style: Emotional warmth compared to Rejection | -0.751 | 0.5528 | -1.835 | 0.332 | 1.848 | 1 | 0.174 |
| Father’s occupation: Not foreign employment | -0.188 | 1.6896 | -3.5 | 3.123 | 0.012 | 1 | 0.911 |
| Mother’s occupation: Not foreign employment | 0.292 | 0.385 | -0.462 | 1.047 | 0.577 | 1 | 0.448 |
| Mother’s level of education: More than primary education | -0.074 | 0.3807 | -0.82 | 0.673 | 0.037 | 1 | 0.847 |
| Baseline: Rosenberg Self-esteem score | 0.64 | 0.043 | 0.555 | 0.724 | 220.996 | 1 | <0.001 |

*Dependent variable: self-esteem level: Rosenberg Self-Esteem scale score at post-intervention*

*Model: Intercept, Arm, Sex, Ethnicity, Religion, Permanent residence, Leadership, School functional type, Attend tuition, Behavioural Inhibition, Anxiety Sensitivity, Mother’s parenting style, Father’s occupation, Mother’s occupation, Mother’s level of education, Baseline: Rosenberg self-esteem scale score*

*Clustering effect is adjusted in GEE by considering clusters as subject while analysis of individual-level data.*

Table S8: Estimates of the Marginal Model for the Effect of the Intervention on Rosenberg’s Self-Esteem Scale Score at the Follow-up, After Adjustment for Confounders and Clustering Effect

| Parameter | Beta | Std. Error | 95% Wald Confidence Interval | | Hypothesis Test | | |
| --- | --- | --- | --- | --- | --- | --- | --- |
|  |  |  | Upper | Lower | Wald  Chi-Square | df | p-value |
| (Intercept) | 12.98 | 2.8755 | 7.344 | 18.616 | 20.377 | 1 | <0.001 |
| **Arm: Study Arm** | **0.435** | **0.3626** | **-0.276** | **1.145** | **1.437** | **1** | **0.231** |
| Sex: Female | 0.314 | 0.3703 | -0.411 | 1.04 | 0.721 | 1 | 0.396 |
| Ethnicity: Non- Sinhala | -0.564 | 1.0176 | -2.559 | 1.43 | 0.307 | 1 | 0.579 |
| Religion: Non- Buddhist | 0.594 | 0.5963 | -0.575 | 1.763 | 0.993 | 1 | 0.319 |
| Permanent residence: Out of Colombo | -0.44 | 0.7121 | -1.836 | 0.956 | 0.382 | 1 | 0.537 |
| Leadership: Never | -0.183 | 0.3172 | -0.805 | 0.438 | 0.334 | 1 | 0.564 |
| School functional type 2 compared to 1AB | -0.346 | 0.4691 | -1.265 | 0.574 | 0.544 | 1 | 0.461 |
| School functional type C compared to 1AB | -1.374 | 0.4445 | -2.245 | -0.503 | 9.552 | 1 | 0.002 |
| Attend tuition: No | 1.085 | 0.6373 | -0.164 | 2.334 | 2.898 | 1 | 0.089 |
| Behavioral Inhibition: High | 0.179 | 0.5676 | -0.933 | 1.292 | 0.1 | 1 | 0.752 |
| Anxiety Sensitivity: High | -0.372 | 0.4176 | -1.191 | 0.447 | 0.793 | 1 | 0.373 |
| Mother’s parenting style: overprotection compared to Rejection | -0.276 | 0.6194 | -1.49 | 0.938 | 0.199 | 1 | 0.656 |
| Mother’s parenting style: Emotional warmth compared to Rejection | -0.597 | 0.7446 | -2.056 | 0.863 | 0.642 | 1 | 0.423 |
| Father’s occupation: Not foreign employment | -0.292 | 1.2185 | -2.68 | 2.096 | 0.057 | 1 | 0.811 |
| Mother’s occupation: Not foreign employment | 0.41 | 0.6882 | -0.939 | 1.758 | 0.354 | 1 | 0.552 |
| Mother’s level of education: More than primary education | -0.686 | 0.6038 | -1.869 | 0.497 | 1.291 | 1 | 0.256 |
| Baseline: Rosenberg Self-esteem score | 0.615 | 0.0775 | 0.463 | 0.767 | 63.049 | 1 | <0.001 |

*Dependent variable: self-esteem level: Rosenberg self-esteem score at follow up*

*Model: Intercept, Arm, Sex, Ethnicity, Religion, Permanent residence, Leadership, School functional type, Attend tuition, Behavioural Inhibition, Anxiety Sensitivity, Mother’s parenting style, Father’s occupation, Mother’s occupation, Mother’s level of education, Baseline: Rosenberg self-esteem score*

*Clustering effect is adjusted in GEE by considering clusters as subject while analysis of individual-level data*
